# Supplementary material for: Immunodominant T-cell epitopes from the SARS-CoV-2 spike antigen reveal robust pre-existing T-cell immunity in unexposed individuals
Source: Sci Rep. 2021 Jun 23;11:13164. doi: 10.1038/s41598-021-92521-4 (PMC8222233; doi:10.1038/s41598-021-92521-4)
Supplement: Supplementary file 4 — Supplementary Information 4. [file 41598_2021_92521_MOESM4_ESM.pdf]

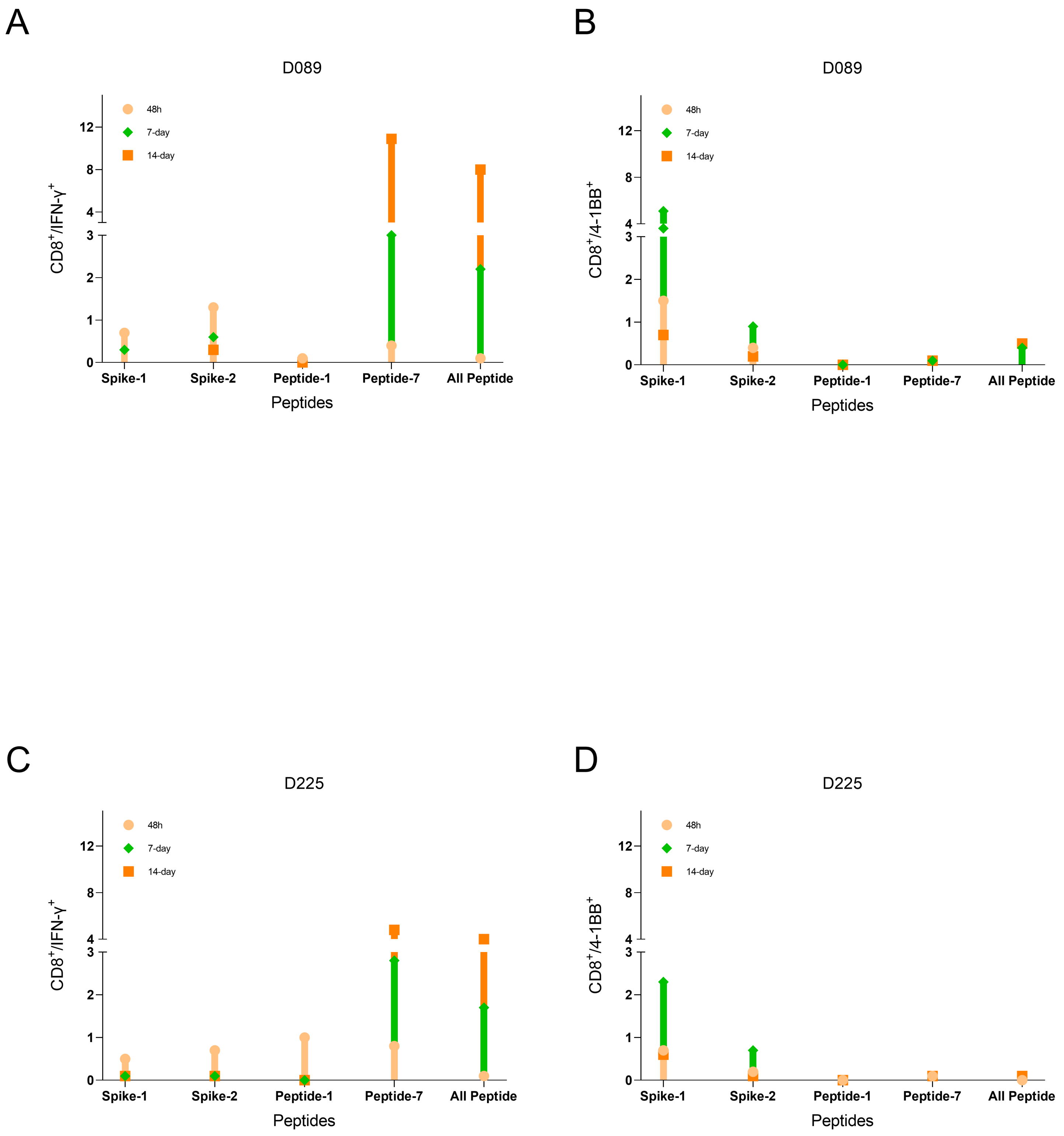

Figure S4. A-D. Kinetics and magnitude of IFN-γ and 4-1BB expression by CD8 T-cells in the presence of Spike-S1, Pep-1, Pep-7, and the All-peptide mix.
