# Supplementary material for: Immunodominant T-cell epitopes from the SARS-CoV-2 spike antigen reveal robust pre-existing T-cell immunity in unexposed individuals
Source: Sci Rep. 2021 Jun 23;11:13164. doi: 10.1038/s41598-021-92521-4 (PMC8222233; doi:10.1038/s41598-021-92521-4)
Supplement: Supplementary file 6 — Supplementary Information 6. [file 41598_2021_92521_MOESM6_ESM.pdf]

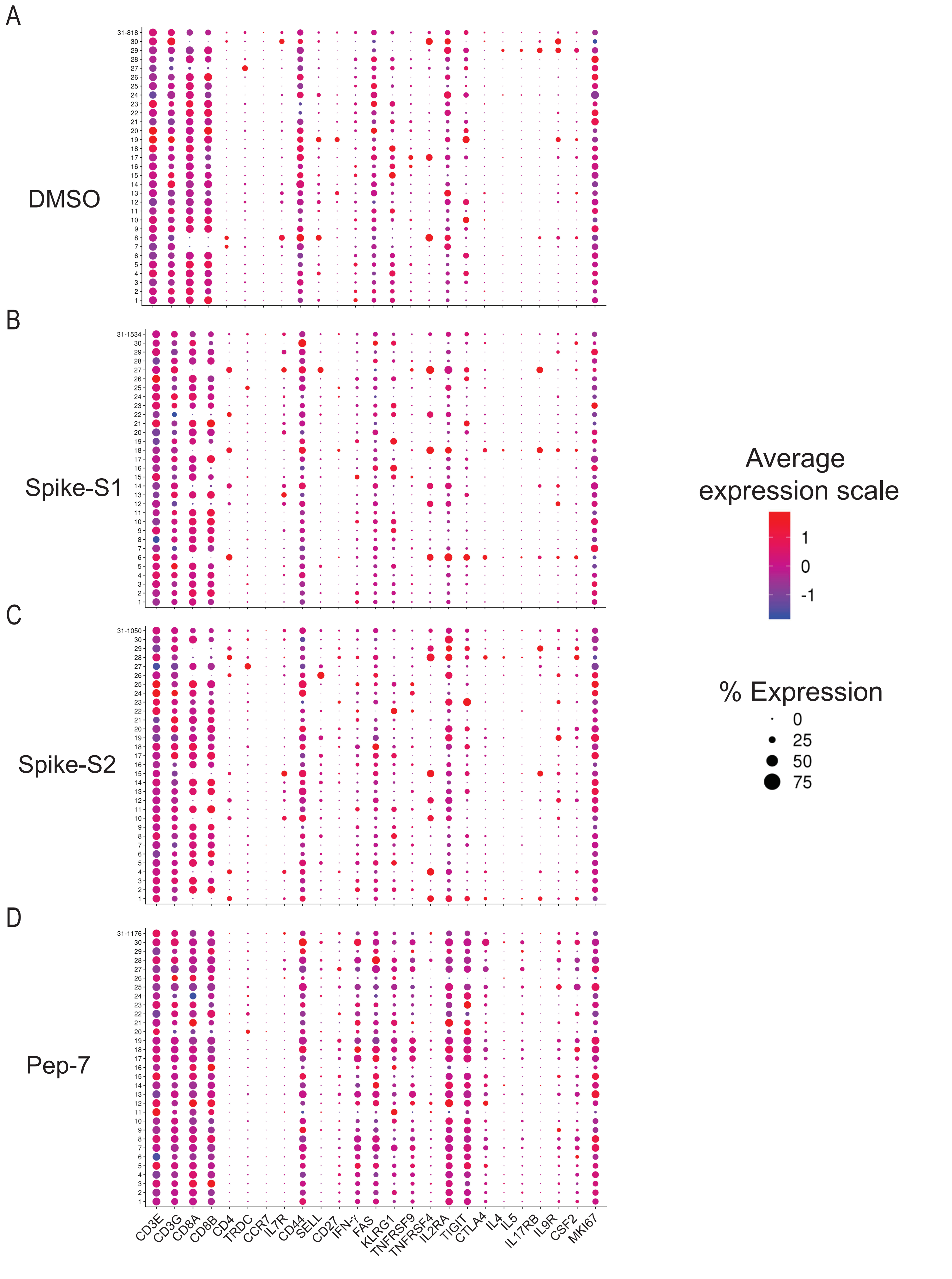

Figure S6. A-D. Expression of T-cell activation and phenotype markers in top-30 clonotypes from single-cell sequencing.
