# Supplementary figures and images for: Immunodominant T-cell epitopes from the SARS-CoV-2 spike antigen reveal robust pre-existing T-cell immunity in unexposed individuals
Source: Sci Rep. 2021 Jun 23;11:13164. doi: 10.1038/s41598-021-92521-4 (PMC8222233; doi:10.1038/s41598-021-92521-4)

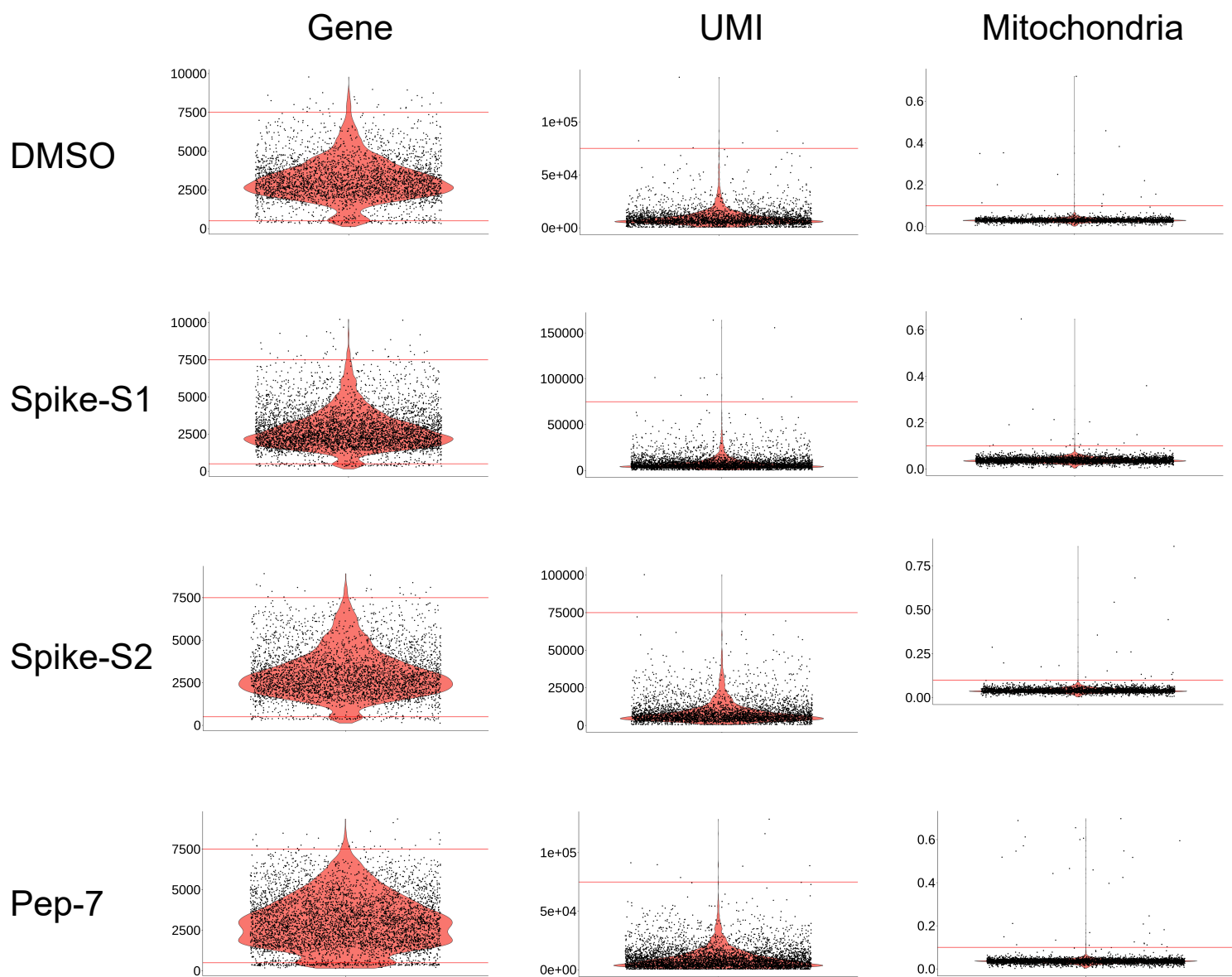

Figure S7. QC of cells from single-cell transcriptomic analysis

Supplement: Supplementary file 7 — Supplementary Information 7. [file 41598_2021_92521_MOESM7_ESM.pdf]

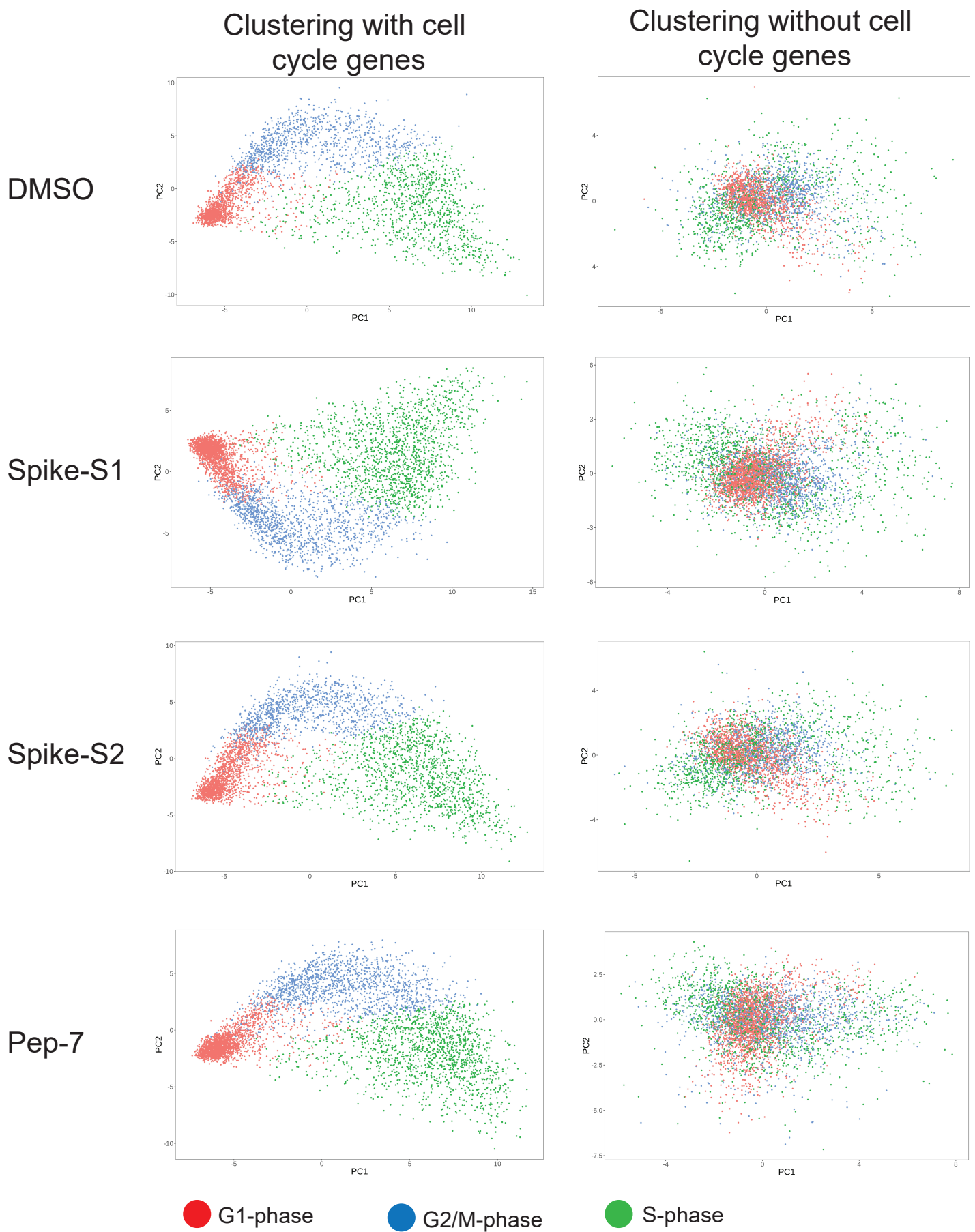

Figure S8. Clustering of cells without and with the expression of G1, G2/M, and S cell-cycle genes.

Supplement: Supplementary file 8 — Supplementary Information 8. [file 41598_2021_92521_MOESM8_ESM.pdf]

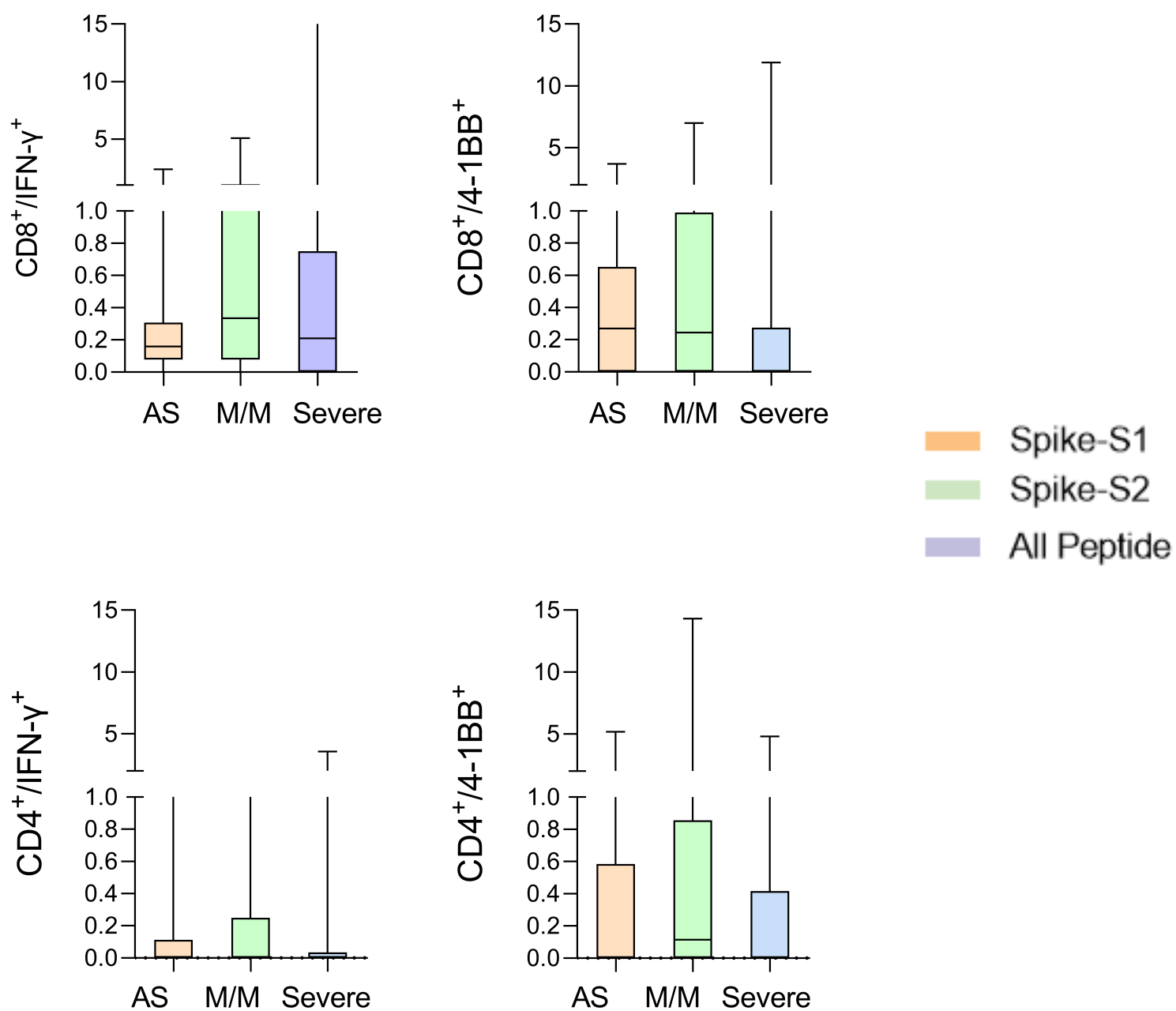

Figure S13. Aggregated T-cell activation data of convalescent patient samples. Related to Figure 5.

Supplement: Supplementary file 13 — Supplementary Information 13. [file 41598_2021_92521_MOESM13_ESM.pdf]
