# Supplementary material for: Immunodominant T-cell epitopes from the SARS-CoV-2 spike antigen reveal robust pre-existing T-cell immunity in unexposed individuals
Source: Sci Rep. 2021 Jun 23;11:13164. doi: 10.1038/s41598-021-92521-4 (PMC8222233; doi:10.1038/s41598-021-92521-4)
Supplement: Supplementary file 9 — Supplementary Information 9. [file 41598_2021_92521_MOESM9_ESM.pdf]

D167

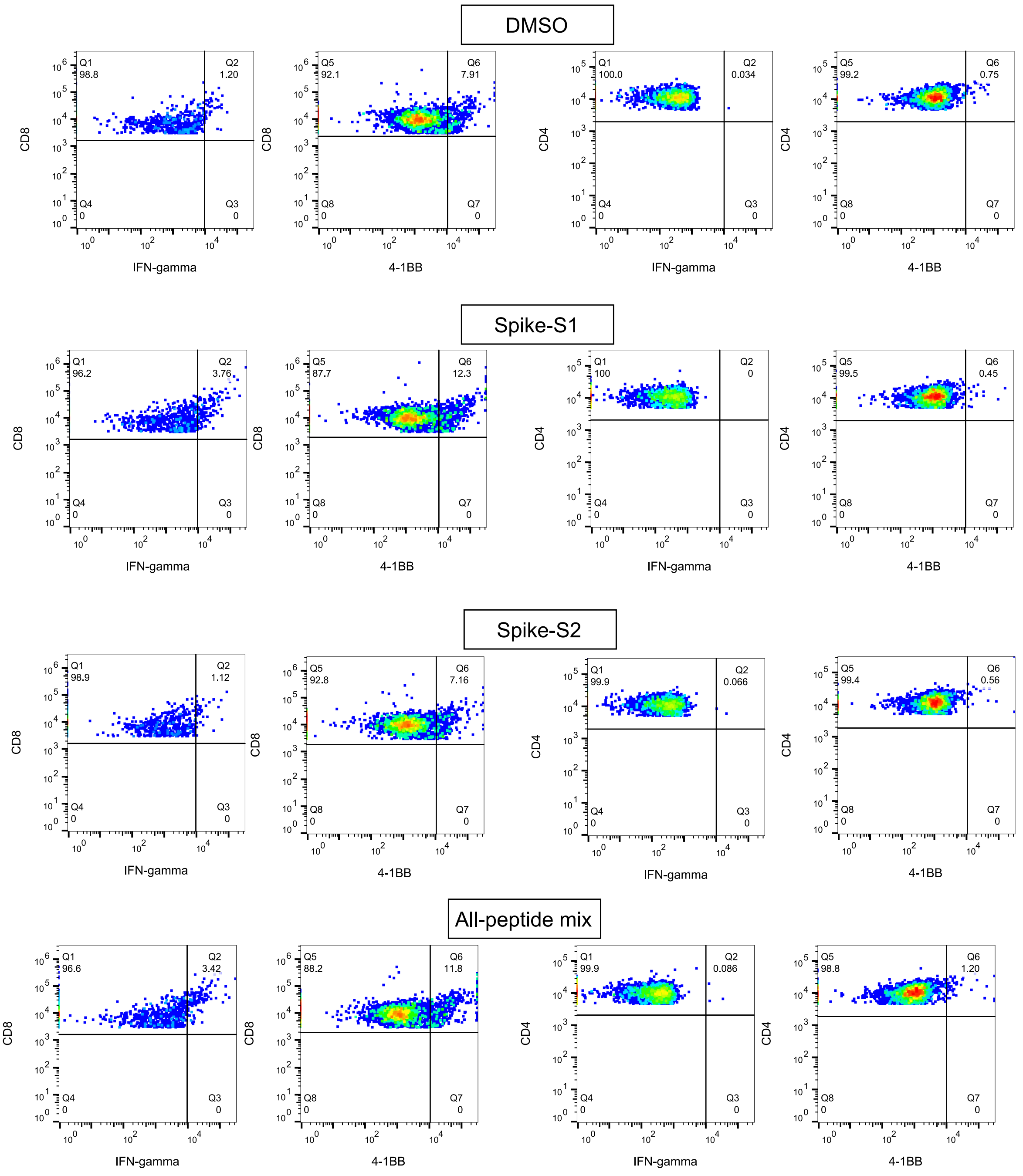

Figure S9. FACS profile of CD4 and CD8 T-cell activation in the presence of different antigens. The gating scheme included the following steps:
Live cells > CD3+ T-cells > CD4 and CD8 T-cells > CD4-IFN-γ/4-1BB and CD8-IFNγ/4-1BB.
