# Supplementary material for: Immunodominant T-cell epitopes from the SARS-CoV-2 spike antigen reveal robust pre-existing T-cell immunity in unexposed individuals
Source: Sci Rep. 2021 Jun 23;11:13164. doi: 10.1038/s41598-021-92521-4 (PMC8222233; doi:10.1038/s41598-021-92521-4)
Supplement: Supplementary file 15 — Supplementary Information 15. [file 41598_2021_92521_MOESM15_ESM.docx]

**Immunodominant T-cell epitopes from the SARS-CoV-2 spike antigen reveal robust pre-existing T-cell immunity in unexposed individuals**

Swapnil Mahajan*^1^, Vasumathi Kode*^2^, Keshav Bhojak*^1^, Coral Karunakaran*^1^, Kayla Lee^2^, Malini Manoharan^1^, Athulya Ramesh^1^, Sudheendra HV^1^., Ankita Srivastava^1^, Rekha Sathian^1^, Tahira Khan^2^, Prasanna Kumar^1^, Ravi Gupta^1^, Papia Chakraborty**^2^ and Amitabha Chaudhuri**^2^

**Table S1. Unexposed Donor characteristics – Related to Figure-2**

| **Sl. No.** | **Donor ID** | **Age** | **Gender** | **HLA class-I and class-II** |
| --- | --- | --- | --- | --- |
| 1 | 116 | 25 | M | A*01:01:01:01/A*68:01:01:02, B*07:06:01/B*15:25:01, C*07:02:01:01/C*07:26:01, DRB1*04:03:01/DRB1*15:01:01:01, DQB1*03:02:01:01/DQB1*06:01:01 |
| 2 | 118 | 30 | M | A*24:02:01:01/A*24:02:01:01, B*07:06:01/B*39:01:01:03, C*07:02:01:01/C*12:04:02, DRB1*01:01:01/DRB1*09:01:02, DQB1*03:03:02:02/DQB1*05:01:01:02 |
| 3 | 122 | 28 | M | A*01:01:01:01/A*33:03:01, B*35:03:01:01/B*52:01:01:01, C*04:01:01:01/C*12:02:02:01, DRB1*14:04:01/DRB1*14:04:01, DQB1*05:03:01:01/DQB1*05:03:01:01 |
| 4 | 132 | 36 | M | A*01:01:01:01/A*31:01:02:01, B*35:03:01:01/B*51:01:01:01, C*04:01:01:01/C*14:07:01, DRB1*13:01:01:01/DRB1*14:07:01, DQB1*05:03:01:01/DQB1*06:03:01 |
| 5 | 142 | 27 | M | A*01:01:01:01/A*01:01:01:01, B*57:01:01/B*57:01:01, C*06:02:01:01/C*06:02:01:01, DRB1*07:01:01:01/DRB1*07:01:01:01, DQB1*03:03:02:01/DQB1*03:03:02:01 |
| 6 | 1610 | 39 | M | A*02:131/A*68:01:02:02, B*07:06:01/B*40:06:01:02, C*07:02:01:01/C*15:07, DRB1*04:03:01/DRB1*15:01:01:01, DQB1*03:02:01:01/DQB1*06:01:01 |
| 7 | 1615 | 41 | M | A*01:01:01:01/A*26:01:01:01, B*52:01:01:01/B*55:01:01, C*01:02:01:01/C*12:02:02:01, DRB1*04:110/DRB1*14:04:01, DQB1*03:02:01:01/DQB1*05:03:01:01 |
| 8 | 167 | 35 | M | A*01:01:01:01, A*02:03:01, B*37:01:01:01/B*51:06:01, C*06:02:01:01/C*14:02:01:01, DRB1*10:01:01:01/DRB1*14:04:01, DQB1*05:01:01:05/DQB1*05:03:01:01 |
| 9 | 169 | 24 | M | A*33:03:01:01/A*33:03:01:01, B*58:01:01:01/B*58:01:01:01, C*03:02:02:01/C*03:02:02:01, DRB1*03:01:01:01/DRB1*13:02:01:01, DQB1*02:01:01/DQB1*06:09:01:01 |
| 10 | 176 | 27 | M | A*01:01:01:01/A*68:01:02:02, B*15:18:01:02/B*51:06:01, C*07:04:01:01/C*14:02:01:01, DRB1*04:01:01:02/DRB1*15:01:01:01, DQB1*03:02:01:01/DQB1*06:01:01 |
| 11 | 089 | 42 | F | A*02:01:01 A*29:02:01, B*40:01:02 B*45:01:01,  C*03:04:01 C*06:02:01 DRB1*07:01:01 DRB1*13:02:01 DRB3*03:01:01 DRB4*01:01:01:01 DQB1*02:02:01 DQB1*06:04:01 DPB1*04:01:01 DPB1*17:01 |
| 12 | 102C | 23 | M | A*11:01 A*24:02 B*07:02 B*40:01 C*03:04 C*07:02 DRB1*13:02 DRB1*15:01DRB3*03:01 DRB5*01:01 DQB1*06:02 DQB1*06:04 DPB1*02:01 |
| 13 | 225 | 42 | M | A*02:01:01 A*30:01:01 B*15:03:01 B*35:01:01 C*04:01:01 C*04:01:01 DRB1*03:01:01 DRB1*13:02:01 DRB3*02:02:01 DRB3*03:01:01 DQB1*02:01:01 DQB1*06:09:01 DPB1*01:01:02 DPB1*04:01:01 |
| 14 | 242 | 46 | M | A*03:01 A*24:02 B*07:02 B*40:01 C*03:04 C*07:02  HLA class-II not available |
| 15 | 384 | 50 | M | A*01:01 A*02:01 B*08:01 B*44:02 C*07:01 C*07:04  HLA class-II not available |
| 16 | 801C | 23 | M | A*11:01 A*29:02 B*38:01 B*44:03 C*12:03 C*16:01 DRB1*04:07  DRB1*07:01 DRB4*01:01 DRB4*01:03 DQB1*02:02 DQB1*03:01 DPB1*04:01 DPB1*04:01 |
| 17 | 907C | 19 | M | A*02:01 A*02:01 B*38:01 B*51:01 C*12:03 C*14:02 DRB1*03:01 DRB1*13:02 DRB3*01:01 DRB3*03:01 DQB1*02:01 DQB1*06:04 DPB1*01:01 DPB1*04:01 |
