# Supplementary material for: Immunodominant T-cell epitopes from the SARS-CoV-2 spike antigen reveal robust pre-existing T-cell immunity in unexposed individuals
Source: Sci Rep. 2021 Jun 23;11:13164. doi: 10.1038/s41598-021-92521-4 (PMC8222233; doi:10.1038/s41598-021-92521-4)
Supplement: Supplementary file 17 — Supplementary Information 17. [file 41598_2021_92521_MOESM17_ESM.docx]

**Immunodominant T-cell epitopes from the SARS-CoV-2 spike antigen reveal robust pre-existing T-cell immunity in unexposed individuals**

Swapnil Mahajan*^1^, Vasumathi Kode*^2^, Keshav Bhojak*^1^, Coral Karunakaran*^1^, Kayla Lee^2^, Malini Manoharan^1^, Athulya Ramesh^1^, Sudheendra HV^1^., Ankita Srivastava^1^, Rekha Sathian^1^, Tahira Khan^2^, Prasanna Kumar^1^, Ravi Gupta^1^, Papia Chakraborty**^2^ and Amitabha Chaudhuri**^2^

**Table S3. The number of cells present in each immune cell cluster and their distribution in different cell types**. For each treatment, the total number of cells sequenced on the 10X platform is shown in parenthesis. The number of cells belonging to each immune cell type is given. T-cell cluster expressed CD3E and CD3G but did not express any markers of CD4 or CD8 T-cells (refer to Figure S3B). ‘Not defined’ cluster could not be assigned to any specific cell type based on marker expression (Figure S3).

| Cluster | DMSO (3399) | Spike-S1 (4991) | Spike-S2 (3965) | Pep-7 (4905) |
| --- | --- | --- | --- | --- |
| CD8 | 2048 | 2452 | 1523 | 3033 |
| CD4 | 252 | 634 | 1102 |  |
| γ/δ | 996 | 1378 | 727 | 827 |
| NKT | 34 | 373 | 540 | 236 |
| T-cell |  | 86 |  | 647 |
| Not defined | 69 | 68 | 73 | 162 |
