# Supplementary material for: Immunodominant T-cell epitopes from the SARS-CoV-2 spike antigen reveal robust pre-existing T-cell immunity in unexposed individuals
Source: Sci Rep. 2021 Jun 23;11:13164. doi: 10.1038/s41598-021-92521-4 (PMC8222233; doi:10.1038/s41598-021-92521-4)
Supplement: Supplementary file 18 — Supplementary Information 18. [file 41598_2021_92521_MOESM18_ESM.docx]

**Immunodominant T-cell epitopes from the SARS-CoV-2 spike antigen reveal robust pre-existing T-cell immunity in unexposed individuals**

Swapnil Mahajan*^1^, Vasumathi Kode*^2^, Keshav Bhojak*^1^, Coral Karunakaran*^1^, Kayla Lee^2^, Malini Manoharan^1^, Athulya Ramesh^1^, Sudheendra HV^1^., Ankita Srivastava^1^, Rekha Sathian^1^, Tahira Khan^2^, Prasanna Kumar^1^, Ravi Gupta^1^, Papia Chakraborty**^2^ and Amitabha Chaudhuri**^2^

**Table-S4. Convalescent patient characteristics – Related to Figure 5.** Blood samples were collected between 45 – 60 days after testing positive for SARS-CoV-2. Individuals with mild fever and minimal respiratory distress syndrome who were admitted to the hospital and kept under observation were classified as mild to moderate. Patients showing severe respiratory distress syndrome and admitted to the ICU were classified as severe.

| **Sl. No.** | **Patient ID** | **Age** | **Gender** | **Diagnosis** | **Severity** | **Hospitalization** |
| --- | --- | --- | --- | --- | --- | --- |
| 1 | CC01 | 27 | M | RT-PCR | Asymptomatic | No |
| 2 | CC02 | 28 | M | RT-PCR | Asymptomatic | No |
| 3 | CC03 | 23 | F | RT-PCR | Asymptomatic | No |
| 4 | CC04 | 23 | F | RT-PCR | Asymptomatic | No |
| 5 | CC05 | 23 | F | RT-PCR | Asymptomatic | No |
| 6 | CC06 | 46 | F | RT-PCR | Asymptomatic | No |
| 7 | CC07 | 53 | F | RT-PCR | Asymptomatic | No |
| 8 | CC08 | 32 | M | RT-PCR | Asymptomatic | No |
| 9 | CC09 | 23 | F | RT-PCR | Mild to Moderate | Yes |
| 10 | CC10 | 49 | F | RT-PCR | Mild to Moderate | Yes |
| 11 | CC11 | 25 | M | RT-PCR | Mild to Moderate | Yes |
| 12 | CC12 | 55 | F | RT-PCR | Mild to Moderate | Yes |
| 13 | CC13 | 57 | M | RT-PCR | Mild to Moderate | Yes |
| 14 | CC14 | 30 | M | RT-PCR | Mild to Moderate | Yes |
| 15 | CC15 | 61 | M | RT-PCR | Severe | ICU |
| 16 | CC16 | 52 | M | RT-PCR | Severe | ICU |
| 17 | CC17 | 53 | M | RT-PCR | Severe | ICU |
| 18 | CC18 | 56 | F | RT-PCR | Severe | ICU |
| 19 | CC19 | 53 | M | RT-PCR | Severe | ICU |
