# Supplementary material for: Immunodominant T-cell epitopes from the SARS-CoV-2 spike antigen reveal robust pre-existing T-cell immunity in unexposed individuals
Source: Sci Rep. 2021 Jun 23;11:13164. doi: 10.1038/s41598-021-92521-4 (PMC8222233; doi:10.1038/s41598-021-92521-4)
Supplement: Supplementary file 19 — Supplementary Information 19. [file 41598_2021_92521_MOESM19_ESM.docx]

**Immunoominant T-cell epitopes from the SARS-CoV-2 spike antigen reveal robust pre-existing T-cell immunity in unexposed individuals**

Swapnil Mahajan*^1^, Vasumathi Kode*^2^, Keshav Bhojak*^1^, Coral Karunakaran*^1^, Kayla Lee^2^, Malini Manoharan^1^, Athulya Ramesh^1^, Sudheendra HV^1^., Ankita Srivastava^1^, Rekha Sathian^1^, Tahira Khan^2^, Prasanna Kumar^1^, Ravi Gupta^1^, Papia Chakraborty**^2^ and Amitabha Chaudhuri**^2^

**Table S5. Cell Culture and FACS reagents.**

| **Reagents used for culturing PBMCs** | | | |
| --- | --- | --- | --- |
| **S.No** | **Reagent** | **Catalog No.** | **Supplier** |
| 1 | Gibco™ RPMI 1640 Medium | 11875085 | Gibco |
| 2 | Human Serum | H4522 | Sigma-Aldrich, USA |
| 3 | Gibco™ Penicillin-Streptomycin (10,000 U/mL) | 15-140-122 | Gibco |
| 4 | Recombinant Human IL-15 (10µg) | 200-15 | Stemcell Technologies |
| 5 | Human Recombinant IL-2 (10µg) | 78036.1 | Stemcell Technologies |

| **Antibodies for T cell diagnostics & activation** | | | |
| --- | --- | --- | --- |
| **PBMC Diagnostic Panel** | | | |
| **S.No** | **Antibody** | **Catalog No.** | **Supplier** |
| 1 | CD8 APC (Clone: SK1) | 17008742 | eBioscence |
| 2 | CD45RO FITC (Clone: UCHL1) | 11045742 | eBioscence |
| 3 | CD56 FITC (Clone: TULY56) | 11056642 | eBioscence |
| 4 | CD57 FITC (Clone: TB01) | 11057742 | eBioscence |
| 5 | CD14 PE (Clone: 61D3) | 12014942 | eBioscence |
| 6 | CD16 PE (Clone: CB16) | 12016842 | eBioscence |
| 7 | CD4 PerCP - C5.5 (Clone: OKT4) | 45004842 | eBioscence |
| 8 | TCR γδ FITC/ TCR γδ BV 421 (Clone: 11F2) | 347903/744870 | BD Biosciences |
| **T cell Activation panel** | | | |
| 9 | CD8 PerCP - C5.5 (Clone: SK1) | 344710 | Biolegend |
| 10 | CD4 V450/CD4 BV510 (Clone: SK3) | 651849/344634 | BD/Biolegend |
| 11 | IFN γ APC (Clone: 4S.B3) | 502512 | Biolegend |
| 12 | TCR γδ FITC/ TCR γδ PE-Cy™7 (Clone: 11F2) | 347903/655410 | BD Biosciences |
| 13 | 41BB PE / 41BB FITC (Clone: 4B4 (4B4-1) | 12-1379-42/11137942 | eBioscence |
