# Supplementary material for: Immunodominant T-cell epitopes from the SARS-CoV-2 spike antigen reveal robust pre-existing T-cell immunity in unexposed individuals
Source: Sci Rep. 2021 Jun 23;11:13164. doi: 10.1038/s41598-021-92521-4 (PMC8222233; doi:10.1038/s41598-021-92521-4)
Supplement: Supplementary file 20 — Supplementary Information 20. [file 41598_2021_92521_MOESM20_ESM.docx]

**Immunodominant T-cell epitopes from the SARS-CoV-2 spike antigen reveal robust pre-existing T-cell immunity in unexposed individuals**

Swapnil Mahajan*^1^, Vasumathi Kode*^2^, Keshav Bhojak*^1^, Coral Karunakaran*^1^, Kayla Lee^2^, Malini Manoharan^1^, Athulya Ramesh^1^, Sudheendra HV^1^., Ankita Srivastava^1^, Rekha Sathian^1^, Tahira Khan^2^, Prasanna Kumar^1^, Ravi Gupta^1^, Papia Chakraborty**^2^ and Amitabha Chaudhuri**^2^

**SUPPLEMENTARY FIGURES LEGEND**

**Figure S1**. **A-E.** Kinetics and magnitude of IFN-γ expression by CD8 T-cells in the presence of individual peptides from the 11-peptide-mix in unexposed donors. PBMCs from unexposed donors were incubated with individual peptides for the indicated time points and the magnitude of CD8 T-cell activation was quantitated by intracellular IFN-γ staining by FACS. See Methods for more details.

**Figure S2.** **A-F.** Kinetics and magnitude of 4-1BB expression by CD8 T-cells in the presence of the 11-peptide mix or individual peptides in unexposed donors. See Figure S1 legend and Methods for more details. A. Activation of CD8 T-cells in different donors. Representative FACS plots of donors D142, D167, D169, and D176 are given in Figure S9-S12.

**Figure S3. A-H.** Kinetics and magnitude of IFN-γ and 4-1BB expression by CD4 T-cells in the presence of individual peptides in unexposed donors. See Figure S1 legend and Methods for more details.

**Figure S4. A-D.** Kinetics and magnitude of IFN-γ and 4-1BB expression by CD8 T-cells in the presence of Spike-S1, Pep-1, Pep-7, and the All-11-peptide mix. A. PBMCs from donor D089 were incubated with the indicated antigens for different time points and the expression of IFN-γ and 4-1BB in CD8 T-cells was quantitated by FACS. B. Same for donor D225.

Cells were used for bulk and single-cell TCR/transcriptomic analysis. See text for details

**Figure S5.** **A-D.** Expression of T-cell activation and phenotype markers in different cell clusters from single-cell sequencing. Expression of cell-type-specific and T-cell phenotype markers are shown on the X-axis. The color and the size of the circles indicate average expression and the proportion of cells expressing a particular marker respectively.

**Figure S6.** **A-D.** Expression of T-cell activation and phenotype markers in top-30 clonotypes from single-cell sequencing. Expression of cell-type-specific and T-cell phenotype markers in the top 30 clonotypes are shown on the X-axis. Each clonotype corresponds to a cluster of cells with a similar gene expression pattern.

**Figure S7.** QC of cells from single-cell transcriptomic analysis. The cells outside the red bars expressing mitochondrial genes were excluded from the analysis (see Methods for details and Supplementary Methods for the codes.

**Figure S8.** Clustering of cells without and with the expression of G1, G2/M, and S cell cycle genes. Cell-type-specific clustering was achieved by regressing out the expression of cell cycle genes. See Supplementary Methods for the codes.

**Figure S9 – S12.** FACS profile of CD4 and CD8 T-cell activation in the presence of different antigens. The gating scheme included the following steps: Live cells > CD3^+^ T-cells > CD4 and CD8 T-cells > CD4-IFN-g/4-1BB and CD8-IFNg/4-1BB.

**Figure S13.** Aggregated T-cell activation data of convalescent patient samples. PBMCs from asymptomatic (AS), mild/moderate (M/M), and severe COVID-19 infected patients were incubated with the indicated antigens for 48 h. CD4 and CD8 T-cells expressing IFN-γ and/or 4-1BB were quantitated by FACS. Individual patient data is shown in Figure 5.

**Figure S14.** A comparison of CD8 T-cell activation in unexposed donors and convalescent asymptomatic COVID-19 infected patients. CD8 T-cell activation from 48h time point was plotted. See Methods for details
