# Supplementary material for: Immunodominant T-cell epitopes from the SARS-CoV-2 spike antigen reveal robust pre-existing T-cell immunity in unexposed individuals
Source: Sci Rep. 2021 Jun 23;11:13164. doi: 10.1038/s41598-021-92521-4 (PMC8222233; doi:10.1038/s41598-021-92521-4)
Supplement: Supplementary file 21 — Supplementary Information 21. [file 41598_2021_92521_MOESM21_ESM.docx]

**Immunodominant T-cell epitopes from the SARS-CoV-2 spike antigen reveal robust pre-existing T-cell immunity in unexposed individuals**

Swapnil Mahajan*^1^, Vasumathi Kode*^2^, Keshav Bhojak*^1^, Coral Karunakaran*^1^, Kayla Lee^2^, Malini Manoharan^1^, Athulya Ramesh^1^, Sudheendra HV^1^., Ankita Srivastava^1^, Rekha Sathian^1^, Tahira Khan^2^, Prasanna Kumar^1^, Ravi Gupta^1^, Papia Chakraborty**^2^ and Amitabha Chaudhuri**^2^

Supplementary Methods: Single-cell analysis codes

1. **Cell-Ranger Commands**

Single-cell sequencing fastq files were processed using the cell ranger pipeline (10X Genomics).

Gene expression analysis

*cellranger count --id=Sample_GEX_Output --transcriptome=refdata-cellranger-GRCh38-3.0.0 --fastqs=”fastq_files_path” --sample=Sample_GEX*

VDJ analysis

*cellranger vdj --id=Sample-TCR_Output --reference=refdata-cellranger-vdj-GRCh38-alts-ensembl-2.0.0 --fastqs=”fastq_files_path” --sample=Sample-TCR*

**II. Single Cell Analysis Pipeline (Seurat)**

**Config file (input files and parameters for the analysis)**

| **Parameter** | **Value** |
| --- | --- |
| data_dir | Sample_GEX_result/filtered_feature_bc_matrix/ |
| path_src | LIBRARY/ |
| md_file_data | Sample_TCR_result/outs/all_contig_annotations.csv |
| low_nGene | 500 |
| high_nGene | 7500 |
| low_nUMI | 500 |
| high_nUMI | 75000 |
| low_percentMito | 0 |
| high_percentMito | 0.1 |
| low_percentTop50 | 0 |
| high_percentTop50 | 0.5 |
| mean_x_low_cutoff | 0.0125 |
| mean_x_high_cutoff | 3 |
| dispersion_y_cutoff | 0.5 |
| dims_use_pca | 15 |
| cluster_resolution | 0.6 |
| min_pct_markers | 0.25 |
| thresh_use_markers | 0.25 |
| top_genes | 25 |
| markers2plot | ANPEP,"ANXA3","BCL6","CCL3","CCL4" |
| project_name | “Project_Name” |
| data1_alias | Sample6 |

**R Commands**

| ---  title: "ScRNA-seq Cluster Analyses"  output: html_document  params:  config_file: "NoDefaultConfigFile"  out_dir : "NoDefaultOutDir"  ---  [//]: # Load Library & Data  ```{r setup, include=FALSE}  library(Seurat) # This is the main package for our analyses  library(dplyr) # data frame manipulation package  library(Matrix) # converting sparse matrix to regular matrix  library(hash) # this package is needed in various functions for gene annotation  library(DT) # displaying tabular data  library(knitr) # Rmarkdown document creation  library(reshape2) # melt the dataframes for ggplot usage  library(ggsignif) #adds significance values for violin plots and boxplots  library(ggpubr)  library(gridExtra) #arranges multiple graphic objects in a well defined graphic space  knitr::opts_chunk$set(echo = TRUE, fig.path=paste(params$out_dir))  ```  ```{r, loaddata, results='hide',echo=FALSE}  config <- read.table(file=params$config_file, sep = "\t", header = T, stringsAsFactors = F) # read config file from command line. This is a tab separated file.  out_dir <- as.character(params$out_dir)  data = as.character(config[1,2]) # this is the full path name of either 1) 10x data directory with barcodes.tsv, genes.tsv and matrix.mtx files 2) a text file (*.txt) with genes as row names and cells as column names or 3) a seurat R object (*.Robj) file  path_src = as.character(config[2,2]) #full path for the src directory  md_file_data = as.character(config[3,2]) # full path of the metadata file, with cell names as row names and their metadata as columns in a tab separated file  low_nGene = as.numeric(config[4,2]) # cells with number of genes identified below this cutoff will be removed  high_nGene = as.numeric(config[5,2]) # cells with number of genes identified above this cutoff will be removed  low_nUMI = as.numeric(config[6,2]) # cells with read depth below this cutoff will be removed  high_nUMI = as.numeric(config[7,2]) # cells with read depth above this cutoff will be removed  low_percentMito = as.numeric(config[8,2]) # cells with fraction of reads mapping to mitochondrial genes below this cutoff will be removed  high_percentMito = as.numeric(config[9,2]) # cells with fraction of reads mapping to mitochondrial genes above this cutoff will be removed  low_percentTop50 = as.numeric(config[10,2]) # cells with fraction of reads mapping to most highly expressed 50 genes below this cutoff will be removed  high_percentTop50 = as.numeric(config[11,2]) # cells with fraction of reads mapping to most highly expressed 50 genes above this cutoff will be removed  mean_x_low_cutoff = as.numeric(config[12,2]) # for PCA analyses, only genes with mean above this cutoff will be used  mean_x_high_cutoff = as.numeric(config[13,2]) # for PCA analyses, only genes with mean below this cutoff will be used  dispersion_y_cutoff = as.numeric(config[14,2]) # for PCA analyses, only genes with dispersion this cutoff will be used  dims_use_pca = as.numeric(config[15,2]) # number of PCA dimensions to be used for tSNE analyses  cluster_resolution = as.numeric(config[16,2]) # Value of the resolution parameter, use a value above (below) 1.0 if you want to obtain a larger (smaller) number of communities (see ?FindClusters)  min_pct_markers = as.numeric(config[17,2]) #only test genes for differential expression that are detected in a minimum fraction of min.pct cells in either of the two populations (see ?FindAllMarkers)  thresh_use_markers = as.numeric(config[18,2]) #Limit testing to genes for differential genes expression which show, on average, at least X-fold difference (log-scale) between the two groups of cells (see ?FindAllMarkers)  top_genes = as.numeric(config[19,2]) # number of top differentially overexpressed genes to look for marker genes  markers2plot = strsplit(config[20,2],",")[[1]] # list of genes to be plotted on tSNE plots and dotplots  project_name = config[21,2]  markers_table <- read.table(file= paste0(path_src,"/markers.txt"),sep ="\t", header=T) # list of marker genes markers file (markers.txt)  sample <- as.character(config[24,2])  source(paste0(path_src,"/markers.r")) # markers.r is used to label clusters based on marker genes  source(paste0(path_src,"/gene2goid.r")) # gene2goid.r is used to get GO information of marker genes  source(paste0(path_src,"/gene2header.r")) # gene2header.r is used to get annotation for the marker genes  ###################################################################  #Load data for scRNA-seq data analyses.  if (grepl("RData", data)) {  load(data1)  } else if (grepl("txt", data)) {  pbmc.data <- read.table(data, sep ="\t")  pbmc <- CreateSeuratObject(raw.data = pbmc.data, min.cells = 3, project = sample) # create seurat object from text file  rm(pbmc.data)  } else {  pbmc.data <- Read10X(data.dir = data)  pbmc <- CreateSeuratObject(raw.data = pbmc.data, min.cells = 3, project = "S1")  rm(pbmc.data) # remove input data to free up memory  }  if(md_file_data != "NONE"){  md <- read.csv(file=md_file_data)  md <- md[,c(1,17)]  md <- unique(md)  md[,1] <- sub(pattern = "-1", replacement = "", md[,1])  md <- md[md$barcode %in% pbmc@cell.names,]  md_temp <- data.frame("barcode"=as.character(pbmc@cell.names[!(pbmc@cell.names %in% md$barcode)]), "raw_clonotype_id"="None")  md <- rbind(md, md_temp)  md <- md[order(md$barcode),]  md$clonotype <- ""  md[md[,2]=="clonotype1",3] <- paste(sample,"_","clonotype1", sep="")  md[md[,2]=="clonotype2",3] <- paste(sample,"_","clonotype2", sep="")  md[md[,2]=="clonotype3",3] <- paste(sample,"_","clonotype3", sep="")  md[md[,3]=="",3] <- paste(sample,"_","other", sep="")  md <- md[,-2]  row.names(md) <- md[,1]  md_n <- ncol(md)  } else {md_n <- 0}  if (md_n > 0) {  for (i in 1:md_n) {  pbmc <- AddMetaData(object = pbmc, metadata = md[i], col.name = colnames(md)[i])  }  }  ```  [//]: # QC  ## QC plots  These plots indicate common QC properties like distribution of 1) number of reads per cell, 2) number of genes per cell, 3) fraction of reads mapping to mitochondrial genes detected per cell (higher fraction suggests cell disruption), 4) fraction of reads mapping to top 50 expressed genes, 5) and correlation between read depth and number of genes identified per cell.  ``````{r,qc,fig.height=7,fig.width=13, echo=FALSE}  print ("Number of genes Number of cells")  print (dim(pbmc@data)) # number of genes and cells before QC  mito.genes <- grep(pattern = "^MT-", x = rownames(x = pbmc@raw.data), value = TRUE) # identify mitochondrial genes (gene names starting with MT)  percent.mito <- Matrix::colSums(pbmc@raw.data[mito.genes, ])/Matrix::colSums(pbmc@raw.data) # calculate fraction of reads mapping to mitochondrial genes  pbmc <- AddMetaData(object = pbmc, metadata = percent.mito, col.name = "percent.mito") # add the percent.mito metadata  total_reads <- as.data.frame(cbind(Matrix::rowSums(pbmc@raw.data),Matrix::rowSums(pbmc@raw.data))) # add total reads for each genes across all cells  top50 <- rownames(total_reads[order(total_reads$V1,decreasing = TRUE),][1:50,]) #sort genes based on total reads and take top 50 genes  percent.top50 <- Matrix::colSums(pbmc@raw.data[top50, ])/Matrix::colSums(pbmc@raw.data) # identify the fraction of reads mapping to top50 genes for each cell  pbmc <- AddMetaData(object = pbmc, metadata = percent.top50, col.name = "percent.top50") # add the percent.top50 metadata  VlnPlot(object = pbmc, features.plot = c("nUMI", "nGene", "percent.mito","percent.top50"), nCol = 4) #create violin plot before QC filtering  pbmc <- FilterCells(object = pbmc, subset.names = c("nGene", "nUMI","percent.mito", "percent.top50"), low.thresholds = c(low_nGene, low_nUMI, low_percentMito, low_percentTop50), high.thresholds = c(high_nGene, high_nUMI, high_percentMito, high_percentTop50)) # filter cells based on input parameters  rm(percent.mito,percent.top50, total_reads,top50) # remove data to free up memory  ```  ## After QC  ``````{r , postqc,fig.height=7,fig.width=13, echo=FALSE}  print ("Number of genes Number of cells")  print (dim(pbmc@data))  VlnPlot(object = pbmc, features.plot = c("nUMI", "nGene", "percent.mito","percent.top50"), nCol = 4) #create violin plot after QC  ```  ## nGene-nUMI correlation plot  ```{r , geneplot,fig.height=7,fig.width=13, echo=FALSE}  par(mfrow = c(1, 2))  GenePlot(object = pbmc, gene1 = "nUMI", gene2 = "nGene", cex.use = 0.5) # plot gene vs nUMI dot plot  ```  [//]: # Normalization, finding variable genes, PCA, scaling of the data and Cell cycle gene analysis  ## PCA plot  ```{r, normalize, echo=FALSE}  pbmc <- NormalizeData(object = pbmc, normalization.method = "LogNormalize", scale.factor = 10000) # "LogNormalize" normalizes the gene expression measurements for each cell by the total expression, multiplies this by a scale factor (10,000), and log-transforms the result.  pbmc <- FindVariableGenes(object = pbmc, mean.function = ExpMean, dispersion.function = LogVMR, x.low.cutoff = mean_x_low_cutoff, x.high.cutoff = mean_x_high_cutoff, y.cutoff = dispersion_y_cutoff, do.plot = FALSE) #calculates the average expression and dispersion for each gene, and identifies variable genes to be used later in PCA analyses  print("Number of variable genes")  print(length(x = pbmc@var.genes))  ```  ## PCA analysis  ```{r, pca, results='hide', echo=FALSE}  print ("Start - Cell Cycle genes scoring and Regression")  pbmc <- CellCycleScoring(object = pbmc, s.genes = cc.genes$s.genes, g2m.genes = cc.genes$g2m.genes,set.ident=TRUE)  head(x = pbmc@meta.data)  RidgePlot(object = pbmc, features.plot = c("PCNA", "TOP2A", "MCM6", "MKI67"))  pbmc <- ScaleData(object = pbmc, display.progress = FALSE)  pbmc <- RunPCA(object = pbmc, pc.genes = c(cc.genes$s.genes, cc.genes$g2m.genes), do.print = FALSE)  PCAPlot(object = pbmc)  if (length(levels(pbmc@ident)) > 1000) { #regress out cell-cell variation in gene expression driven by batch ("ident"), read depth and percent.mito  pbmc <- ScaleData(object = pbmc, vars.to.regress = c("S.Score", "G2M.Score","nUMI", "percent.mito", "ident"))  } else { pbmc <- ScaleData(object = pbmc, vars.to.regress = c("S.Score", "G2M.Score","nUMI", "percent.mito"),model.use="linear") }  pbmc <- RunPCA(object = pbmc, pc.genes = pbmc@var.genes, genes.print = 10)  pbmc <- RunPCA(object = pbmc, pc.genes = c(cc.genes$s.genes, cc.genes$g2m.genes), do.print = FALSE)  PCAPlot(object = pbmc)  print ("End - Cell Cycle genes scoring and Regression")  pbmc <- RunPCA(object = pbmc, pc.genes = pbmc@var.genes, do.print = FALSE, pcs.print = 1:5,genes.print = 5) # Run PCA with variable genes previously identified  PCHeatmap(object = pbmc, pc.use = 1:6, cells.use = 500, do.balanced = TRUE,label.columns = FALSE, use.full = FALSE)  ```  List of genes whose expression shows high correlation with different top PCs are shown. One should be careful if a PC shows high expression with housekeeping genes (e.g. ribosomal/mitochondrial) expression.  ```{r, pca_correlations, echo=FALSE}  ngene_numi_pc <- cbind(pbmc@meta.data$nGene,pbmc@meta.data$nUMI,pbmc@dr$pca@cell.embeddings) # for each cell(sample) take out nGene, nUMI and PC values  colnames(ngene_numi_pc)[c(1,2)] <- c("nGene", "nUMI")  print ("correlation of PC values with nUMI and nGene")  cor_matrix <- t(signif(cor(ngene_numi_pc)[c(1,2),],2)) # correlation of nUMI and nGene with PC values  print (cor_matrix)  ```  ## PCA elbow plot  ```{r, elbow,echo=FALSE}  PCElbowPlot(object = pbmc)  ```  PC Elbow plot can be used to identify most informative PCs to be used for t-SNE analyses.  [//]: # tSNE & UMAP analysis  ## tSNE plots  ```{r, tsne_unlabelled,results='hide', echo=FALSE}  pbmc <- FindClusters(object = pbmc, reduction.type = "pca", dims.use = 1:dims_use_pca,resolution = cluster_resolution, print.output = 1, save.SNN = TRUE) # find clusters using PC values  pbmc <- RunTSNE(object = pbmc, dims.use = 1:dims_use_pca, do.fast = TRUE) # display clusters using tSNE plots  number_clusters <- length(unique(pbmc@ident)) # count number of clusters identified  pbmc.markers <- FindAllMarkers(object = pbmc, only.pos = TRUE, min.pct = min_pct_markers, thresh.use = thresh_use_markers) # find differentially overexpressed genes in each cluster  ```  ```{r, tsne_labelled,results='hide', echo=FALSE}  top_overexpressed<-pbmc.markers %>% group_by(cluster) %>% top_n(top_genes, avg_logFC) %>% print(n=number_clusters*top_genes) # Find top ~20 overexpressed genes in each cluster  diff <- setdiff(levels(top_overexpressed$cluster),as.character(unique(top_overexpressed$cluster)))  if(length(diff)!=0)  {  for(i in 1: length(diff)) {  line <- top_overexpressed[1,]  line$cluster <- as.factor(diff[i])  line$gene <- "Dummy_Gene"  top_overexpressed <- bind_rows(top_overexpressed,line)  }  }  top_overexpressed2<-cbind(top_overexpressed$cluster,top_overexpressed$gene) #select only cluster number and gene column  labelled_clusters <- getClusterLabels(top_overexpressed2) # get the clusters labels using getClusterLabels function. This is a custom function  if(length(diff)!=0) {  current.cluster.ids <- as.character(labelled_clusters[,1]) #obtain current cluster Ids  } else {  current.cluster.ids <- as.character(as.numeric(labelled_clusters[,1])-1)  }  new.cluster.ids <- as.character(labelled_clusters[,2]) #obtain new cluster Ids  TSNEPlot(object = pbmc, do.return = TRUE, no.legend = TRUE, do.label = TRUE) # plot tSNE unlabelled clusters  print ("UMAP1")  #jpeg("UMAP1_Labeled.jpg",width=14, height=8, units="in", res=600)  pbmc <- RunUMAP(pbmc, dims = 1:15,force.recalc = T)  #dev.off()  print ("UMAP2")  pbmc <- RunUMAP(pbmc, dims = 1:15,force.recalc = T,metric="euclidean")  #jpeg("UMAP2_Labeled.jpg",width=14, height=8, units="in", res=600)  DimPlot(object = pbmc, reduction = 'umap')  #dev.off()  ```  ```{r,clustermarkers2_3,fig.width=17, echo=FALSE}  markers <- c("CCR7","CD14","LYZ","CD163","CD68","CSF1R","CD8A","CD8B","FAP","THY1","DCN","COL1A1","COL1A2","COL6A1","COL6A2","COL6A3","FCER1A","CST3","FCGR3A","MS4A7","FOXP3","GNLY","NKG7","HSP90AB1","IL7R","MS4A1","PECAM1","VWF","CDH5","CD34","PPBP","TPSAB1","KIT","CPA3") #list of marker genes used  markers_found <- (as.character(top_overexpressed$gene) %in% markers)*1 # find the intersection of marker genes listed above which are actually found as top differentially overexpressed genes in the clusters  markers_found2 <- unique(c(intersect(as.character(top_overexpressed$gene),markers),markers2plot)) # add the makers listed in config file to markers_found variable  kb <- unlist(pbmc@data@Dimnames[1])  markers_found3 <- sort(intersect(markers_found2,kb))  # Plots and differential expression  DotPlot(object = pbmc, genes.plot = as.factor(markers_found3),x.lab.rot=TRUE,plot.legend=TRUE,cols.use=c("blue","red")) #dotplot of marker genes unlabelled  DoHeatmap(object = pbmc, genes.use = as.factor(markers_found3), slim.col.label = TRUE, remove.key = TRUE) # Heatmap of marker genes unlabelled    ```  ```{r, tsne_labelled_1,results='hide', echo=FALSE}  if (md_n > 0) {  for (i in 2:md_n) {  TSNEPlot(pbmc, group.by = colnames(md)[i], colors.use=c("#9ACD32AF","#9932CCAF","#FF1493AF","#CCCCCC7D")) # plot tsne with clusters label foreach column of the metadata file  }  } else {  for (i in 2:md_n) {  TSNEPlot(pbmc, group.by = colnames(md1)[i], colors.use=c("#9ACD32AF","#9932CCAF","#FF1493AF","#CCCCCC7D","#FFFF00AF","#000080AF","#000000AF","#CCCCCC7D")) # plot tsne with clusters label foreach column of the metadata file  }  }  ```  ```{r,clusterIdent, echo=FALSE}  print ("Number of cells in different clusters:")  summary(pbmc@ident)  print ("Following marker genes were used for cluster labelling")  datatable(markers_table,options = list(pageLength = 20)) #print marker table from markers.txt file  ```  ## Expression of markers genes    ```{r,clustermarkers2_1,fig.width=17, echo=FALSE}  DotPlot(object = pbmc, genes.plot = as.factor(markers_found3),x.lab.rot=TRUE,plot.legend=TRUE,cols.use=c("blue","red")) #dotplot of marker genes labelled  DoHeatmap(object = pbmc, genes.use = as.factor(markers_found3), slim.col.label = TRUE, remove.key = TRUE) # Heatmap of marker genes labelled  ```  ```{r,clustermarkers2_2, echo=FALSE}  j = 1;  for (i in 1:ceiling(length(markers_found3)/ 4)) { # show marker gene expression on tSNE plot for each marker gene, with each plot having maximum of 4 marker genes  if (j+3 < length(markers_found3)) {  k = j+3  } else { k = length(markers_found3) }  FeaturePlot(object = pbmc, features.plot = markers_found3[j:k] , cols.use = c("grey", "blue"), reduction.use = "umap",pt.size = .7, pch.use =16)  j = j+4  }  ```  ## Genes differentially expressed in different clusters  ```{r,clustermarkers3, echo=FALSE}  headers <- gene annotation(top_overexpressed$gene) # get gene annotation about the marker gene using a custom function  go <- gene2go(top_overexpressed$gene) # get GO annotation about the marker gene using a custom function  marker_table <- cbind(as.character(top_overexpressed$cluster),as.character(top_overexpressed$gene), headers, go, markers_found) # create a dataframe  colnames(marker_table) <- c("ClusterId", "Gene Name", "Annotation", "GO", "Markers") # give column names  datatable(marker_table, filter = 'top',options = list( # print the table with known marker genes found highlighted in yellow  columnDefs = list(list(targets = 4, visible = FALSE)),  pageLength = top_genes  )) %>% formatStyle(  'Markers',  target = 'row',  backgroundColor = styleEqual(c(0, 1), c('white', 'yellow'))  )  save.image(file= paste(out_dir,"/","Analysis_Seurat",sample1,".RData",sep=""))  ``` |
| --- |

1. **Wrapper script to run the Seurat pipeline**

| rmarkdown::render("/Path_to_pipeline_folder/SingleCell_Pipeline.Rmd", output_file="/Desired_Path_for_saving_output/desired_name_of_html_output.html", params = list(config_file = "/Path/to_config/config.txt",  out_dir="/Desired_Path_for_saving_output/")) |
| --- |

1. **Commands for manual assignment of clusters**

| # We can label the clusters according to differentially expressed genes, clonotypes or our analysis also. And after the manual labelling, we can make all the plots accordingly.  pident=as.factor(clusters)  names(pident)=cellNames  object1@ident=pident |
| --- |
